# SA4503, A Potent Sigma-1 Receptor Ligand, Ameliorates Synaptic Abnormalities and Cognitive Dysfunction in a Mouse Model of ATR-X Syndrome

**DOI:** 10.3390/ijms19092811

**Published:** 2018-09-18

**Authors:** Kouya Yamaguchi, Norifumi Shioda, Yasushi Yabuki, Chen Zhang, Feng Han, Kohji Fukunaga

**Affiliations:** 1 Department of Pharmacology, Graduate School of Pharmaceutical Sciences, Tohoku University, Sendai 980-8578, Japan; kouya.yamaguchi.r3@dc.tohoku.ac.jp (K.Y.); yabukiy@m.tohoku.ac.jp (Y.Y.); 2 Department of Genomic Neurology, Institute of Molecular Embryology and Genetics, Kumamoto University, Kumamoto 860-0811, Japan; shioda@kumamoto-u.ac.jp; 3 College of Pharmaceutical Sciences, Zhejiang University, Hangzhou 31005, Zhejiang, China; chenzhang@zju.edu.cn; 4 School of Pharmacy, Nanjing Medical University, Nanjing 211166, Jiangsu, China; fenghan169@njmu.edu.cn

**Keywords:** ATR-X syndrome, cognitive function, dendritic spine, sigma-1 receptor, brain-derived neurotrophic factor

## Abstract

α-thalassemia X-linked intellectual disability (ATR-X) syndrome is caused by mutations in *ATRX*. An ATR-X model mouse lacking *Atrx* exon 2 displays phenotypes that resemble symptoms in the human intellectual disability: cognitive defects and abnormal dendritic spine formation. We herein target activation of sigma-1 receptor (Sig-1R) that can induce potent neuroprotective and neuroregenerative effects by promoting the activity of neurotrophic factors, such as brain-derived neurotrophic factor (BDNF). We demonstrated that treatment with SA4503, a potent activator of Sig-1R, reverses axonal development and dendritic spine abnormalities in cultured cortical neurons from ATR-X model mice. Moreover, the SA4503 treatment rescued cognitive deficits exhibited by the ATR-X model mice. We further found that significant decreases in the BDNF-protein level in the medial prefrontal cortex of ATR-X model mice were recovered with treatment of SA4503. These results indicate that the rescue of dendritic spine abnormalities through the activation of Sig-1R has a potential for post-diagnostic therapy in ATR-X syndrome.

## 1. Introduction

α-thalassemia X-linked mental retardation (ATR-X, OMIM Entry #301040) syndrome is caused by *ATRX* mutations [1,2]. ATR-X syndrome is characterized by various clinical manifestations, including severe intellectual disability, facial dysmorphism, genital abnormalities, and epileptic seizures [3]. ATR-X syndrome is very rare, probably with an incidence of less than 1/100,000 live-born males [4]. *ATRX* encodes a protein containing two signature domains. The helicase domain has chromatin remodeling activity and DNA translocaze ability. The ATRX-DNMT3-DNMT3L (ADD) domain binds histone H3 tails at H3K4me0K9me2/3 [5,6,7,8,9]. However, the functions of ATRX protein remain unclear.

Several *ATRX* mutations or low *ATRX* gene expression have been identified in patients with ATR-X syndrome [8,9,10,11]. ATR-X patients with an Arg37Stop (R37X) mutation in exon 2 exhibit mild intellectual disability, which is accompanied by reduced expression of ATRX protein in lymphoblastic cells [12,13]. We previously reported that *Atrx* mutant mice lacking exon 2 (Atrx^ΔE2^ mice) express a mutant protein that corresponds to a variant of an R37X mutation seen in human ATR-X syndrome [14]. Atrx^ΔE2^ mice exhibited an 80% reduction in ATRX protein levels [14,15,16], similar to outcomes observed in 27 individuals with ATR-X syndrome [9]. The mice also show cognitive defects and abnormal dendritic spine formation in the medial prefrontal cortex (mPFC) [15] and, consistent with features of intellectual disability [17,18], show longer and thinner dendritic spines compared with wild-type (WT) mice [15]. 

The morphology of dendritic spines relates closely to the function and plasticity of the synapses. For example, the volume of the spine head is directly proportional to the area of postsynaptic density and the number of postsynaptic receptors, and, hence, the size of synaptic currents and synaptic strength [19,20,21,22]. Such spine anomalies have been reported in several neurological disorders associated with cognitive dysfunction, including Alzheimer’s diseases, schizophrenia, and intellectual disability [17,18,23,24]. 

Activation of the sigma-1 receptor (Sig-1R) can induce potent neuroprotective and neuroregenerative effects by promoting the activity of neurotrophic factors, including brain-derived neurotrophic factor (BDNF) [25,26]. BDNF is an important neurotrophic factor linked with neuronal development, synaptic maturation and neural plasticity [27,28,29]. The activation of BDNF pathways improved learning and memory in intellectual disability model mice, such as Rett syndrome and Fragile-X syndrome [30,31]. Here, we show that Sig-1R activation with SA4503, a potent activator of Sig-1R [32], features potential as a therapeutic intervention for diminished cognitive function in ATR-X syndrome.

## 2. Results

### 2.1. Treatment with SA4503 Reverses Abnormality of Axonal Development and Dendritic Filopodia in Cultured Cortical Neurons from Atrx^ΔE2^ (Atrx Mutant Mice Lacking Exon 2) Mice

**Figure 1:**
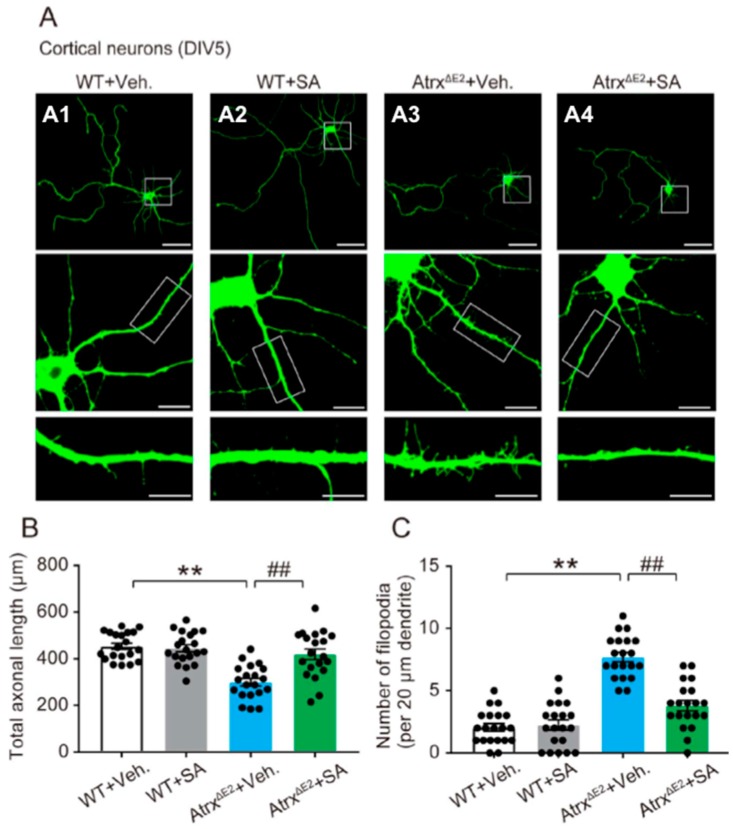
Treatment with SA4503 reverses abnormality of axonal development and dendritic filopodia in cultured Atrx^ΔE2^ (Atrx mutant mice lacking exon 2) neurons. (**A**) Representative images of EGFP-transfected cortical neurons at DIV5. Neurons are stained for anti-GFP. Images in the middle and bottom panels are enlarged from the corresponding boxed areas. Scale bars: top panels, 50 µm; middle panels, 10 µm; bottom panels, 5 µm. (**B**) Total axonal length. ** *p* < 0.01, versus vehicle-treated WT (wild type) neurons; ## *p* < 0.01, versus vehicle-treated Atrx^ΔE2^ neurons by one-way ANOVA with post-hoc Tukey’s test; F (3, 76) = 17.4; *n* = 20 neurons for each group. The experiments were repeated three times with similar results. (**C**) Data show the number of filopodia per 20 µm dendritic length. ** *p* < 0.01, versus vehicle-treated WT neurons; ## *p* < 0.01, versus vehicle-treated Atrx^ΔE2^ neurons by one-way ANOVA with post hoc Tukey’s test; F (3, 76) = 48.26; *n* = 20 neurons for each group. The experiments were repeated three times with similar results. Each bar represents the mean ± SEM. Abbreviations: Veh., vehicle; SA, SA4503.

We investigated changes in synaptic morphology using cultured cortical neurons from Atrx^ΔE2^ mice. To analyze axon development and dendritic filopodia, we labeled cultured neurons by transfection with EGFP at DIV0 and investigated these morphologies at DIV5. We first examined the effect of the *Atrx* gene mutation on the outgrowth of axons (Figure 1A3). Quantification revealed that the lengths of the axons were significantly reduced in Atrx^ΔE2^ compared to those of WT neurons. The decreases of axon length in Atrx^ΔE2^ neurons were significantly restored at 48 h after SA4503 treatment (Figure 1B) (The axon length; WT + vehicle, 453.5 ± 13.3 µm; WT + SA4503, 445.5 ± 15.1 µm; Atrx^ΔE2^ + vehicle, 300.1 ± 14.5 µm; Atrx^ΔE2^ + SA4503, 419.6 ± 22.3 µm). As dendritic spines originate from dendritic filopodia, we hypothesized that Atrx^ΔE2^ neurons change the development of these structures. The number of filopodia was significantly increased in cultured neurons of Atrx^ΔE2^ mice relative to the number measured in WT mice (Figure 1C), and cultured neurons from Atrx^ΔE2^ mice treated with SA4503 showed a significant decrease in the number of filopodia (Figure 1A4,C) (The number of filopodia per 20 µm dendritic length; WT + vehicle, 2.1 ± 0.3; WT + SA4503, 2.1 ± 0.4; Atrx^ΔE2^ + vehicle, 7.7 ± 0.4; Atrx^ΔE2^ + SA4503, 3.8 ± 0.4).

### 2.2. Treatment with SA4503 Ameliorates Dendritic Spine Abnormality in Cultured Cortical Neurons from Atrx^ΔE2^ Mice

**Figure 2:**
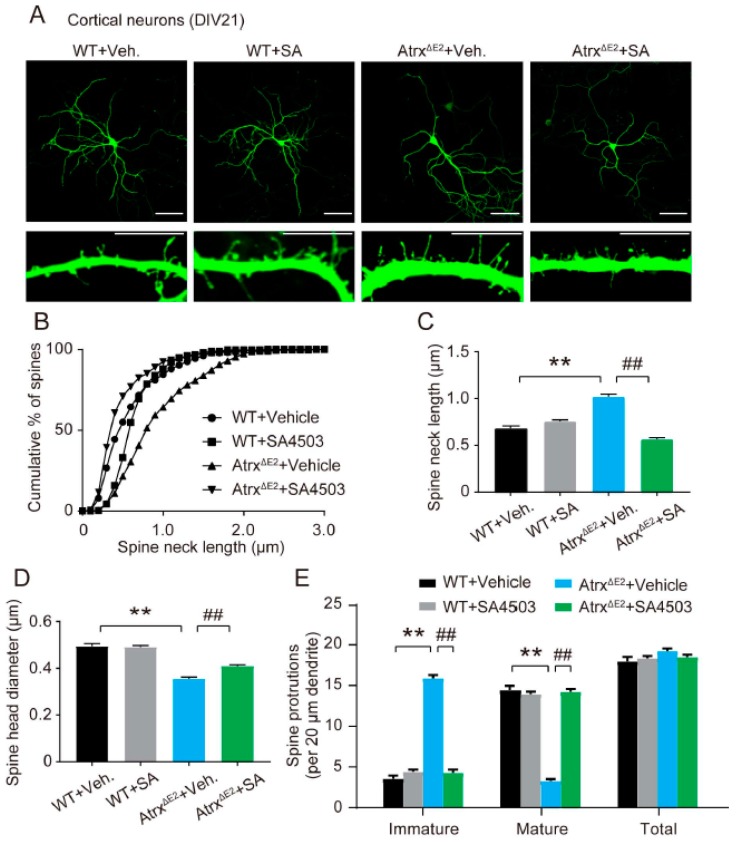
Treatment with SA4503 ameliorates dendritic spine abnormality in cultured Atrx^ΔE2^ neuron. (**A**) Representative images of EGFP-transfected cortical neurons at DIV21. Neurons are stained for anti-GFP. Images in the bottom panels are enlarged from each dendrite. Scale bars: top panels, 100 µm; bottom panels, 10 µm. (**B**) Relationship between cumulative percentage of spines and spine length. *p* < 0.01 in WT versus Atrx^ΔE2^ neurons and in Atrx^ΔE2^ versus SA4503-treated Atrx^ΔE2^ neurons by the Kolmogorov–Smirnov test. (**C**) Data show the number of spines. ** *p* < 0.01 versus vehicle-treated WT neurons; ## *p* < 0.01, versus vehicle-treated Atrx^ΔE2^ neurons by one-way ANOVA with post hoc Tukey’s test; F (3, 1494) = 89.38. (**D**) Data show the spine-head diameter. ** *p* < 0.01 versus vehicle-treated WT neurons; ## *p* < 0.01, versus vehicle-treated Atrx^ΔE2^ neurons by one-way ANOVA with post hoc Tukey’s test; F (3, 76) = 76.38. (**E**) Spine protrusions per 20 µm dendritic length. ** *p* < 0.01 versus vehicle-treated WT neurons; ## *p* < 0.01, versus vehicle-treated Atrx^ΔE2^ neurons by two-way ANOVA with post hoc Bonferroni’s test; F (3, 228) = 2.549, *p* = 0.0566 (group); F (2, 228) = 1008, *p* < 0.01 (spine protrusions), F (6, 228) = 243.6, *p* < 0.01 (interaction between group and spine protrusions); *n* = 20 neurons for each group. The experiments were repeated three times with similar results. WT, *n* = 360 spines; SA4503-treated WT, *n* = 367 spines; Atrx^ΔE2^, *n* = 384 spines; SA4503-treated Atrx^ΔE2^, *n* = 370 spines. Each bar represents the mean ± SEM. Abbreviations: Veh., vehicle; SA, SA4503.

To investigate synaptogenesis, EGFP-transfected cortical neurons were harvested at DIV21. These neurons were fixed and stained with anti-GFP. We then performed spine morphological analysis (Figure 2A). In cultured neurons of Atrx^ΔE2^ mice, spines resembling filopodia were abnormally thin and long relative to those observed in WT neurons. The spine length distribution was shifted to the right in Atrx^ΔE2^ compared to WT neurons, indicating a higher proportion of long and filopodia-like spines in the former. Moreover, SA4503-treated Atrx^ΔE2^ neurons restored the spine abnormality (Figure 2B,C) (spine length; WT + vehicle, 0.68 ± 0.01 µm; WT + SA4503, 0.76 ± 0.01 µm; Atrx^ΔE2^ + vehicle, 1.02 ± 0.01 µm; Atrx^ΔE2^ + SA4503, 0.56 ± 0.01 µm). The spine-head diameter was significantly decreased in Atrx^ΔE2^ compared to WT neurons, while SA4503 treatment ameliorated the decrease observed in the former (Figure 2D) (The spine-head diameter; WT + vehicle, 0.49 ± 0.01 µm; WT + SA4503, 0.49 ± 0.01 µm; Atrx^ΔE2^ + vehicle, 0.36 ± 0.01 µm; Atrx^ΔE2^ + SA4503, 0.41 ± 0.01 µm). As expected, Atrx^ΔE2^ neurons showed increased numbers of immature-appearing and fewer mature-appearing spines than did their WT counterparts. SA4503 treatment in Atrx^ΔE2^ neurons significantly reduced the number of immature spines and increased the number of mature spines, without changing total spine numbers (Figure 2E). (The number of mature spines per 20 µm dendritic length: WT + vehicle, 14.4 ± 0.5; WT + SA4503, 13.9 ± 0.3; Atrx^ΔE2^ + vehicle, 3.3 ± 0.2; Atrx^ΔE2^ + SA4503, 14.2 ± 0.4. The number of immature spines per 20 µm dendritic length: WT + vehicle, 3.6 ± 0.4; WT + SA4503, 4.4 ± 0.3; Atrx^ΔE2^ + vehicle, 15.9 ± 0.4; Atrx^ΔE2^ + SA4503, 4.3 ± 0.4.)

### 2.3. Treatment with SA4503 Rescues Memory and Cognitive Deficits Seen in Atrx^ΔE2^ Mice

**Figure 3:**
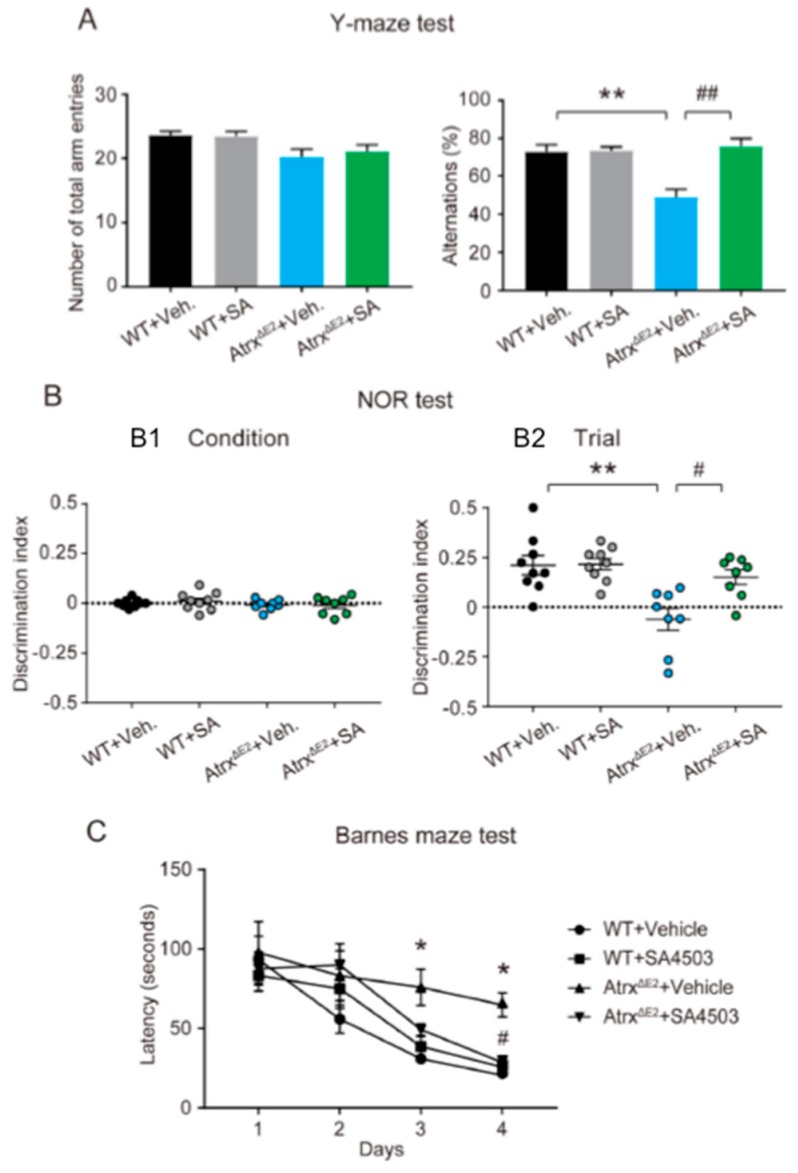
SA4503 treatment decreased cognitive deficits in Atrx^ΔE2^ mice based on memory-related tests. (**A**) Number of total arm entries (left) and alternations (right) in a Y-maze test. ** *p* < 0.01, versus vehicle-treated WT mice; ## *p* < 0.01, versus vehicle-treated Atrx^ΔE2^ mice by one-way ANOVA with post hoc Tukey’s test; F (3, 18) = 12.98. (**B**) Novel object recognition task. Discrimination index of object exploration during the sample phase (left), and while exploring familiar and new objects in the test phase after 24 h (right). ** *p* < 0.01, versus vehicle-treated WT mice; # *p* < 0.05, versus vehicle-treated Atrx^ΔE2^ mice by one-way ANOVA with post hoc Tukey’s test; F (3, 30) = 8.898. (**C**) Barnes maze test measurement of total latency (s) to reach the target hole. * *p* < 0.05 by two-way ANOVA with post hoc Bonferroni’s test; F (3, 120) = 6.535, *p* < 0.01 (group); F (3, 120) = 25.16, *p* < 0.01 (day), F (9, 120) = 1.184, *p* = 0.3112 (interaction between group and day). Each bar represents the mean ± SEM. Abbreviations: Veh., vehicle; SA, SA4503. WT + Vehicle: *n* = 9 mice, WT + SA4503: *n* = 9 mice, Atrx^ΔE2^ + Vehicle: *n* = 8 mice, Atrx^ΔE2^ + SA4503: *n* = 8 mice.

In the Y-maze test, measurement of impairment was based on the percentage of alternation behaviors relative to WT mice without a change in the total number of arm entries. SA4503 treatment increased the percentage of spontaneous alternation behaviors in Atrx^ΔE2^ mice (Figure 3A) (the percentage of alternation behaviors: WT + vehicle, 73.2 ± 3.5; WT + SA4503, 73.7 ± 1.9; Atrx^ΔE2^ + vehicle, 49.1 ± 4.0; Atrx^ΔE2^ + SA4503, 75.9 ± 4.0). In the novel object recognition task, we observed no differences in the discrimination index using the same object across training trials in all groups (Figure 3B1). After a 24 h retention interval between the trial and test sessions, Atrx^ΔE2^ mice revealed a significantly lower discrimination index for novel objects than did WT mice. The discrimination index of Atrx^ΔE2^ mice treated with SA4503 was significantly higher than that of vehicle-treated Atrx^ΔE2^ groups (Figure 3B2) (the discrimination index in test session: WT + vehicle, 0.21 ± 0.05; WT + SA4503, 0.22 ± 0.03; Atrx^ΔE2^ + vehicle, −0.06 ± 0.06; Atrx^ΔE2^ + SA4503, 0.15 ± 0.04). In the Barnes maze test, WT mice featured a significant decline in latency time to enter the target hole across the four days of training, indicating a normal improvement of cognitive performance. By contrast, the cognitive performance of Atrx^ΔE2^ mice did not improve, because longer latency times was required compared to WT mice. SA4503 administration significantly rescued the latency time (Figure 3C) (the latency time in Day 4: WT + vehicle, 20.6 ± 1.0 s; WT + SA4503, 25.7 ± 3.1 s; Atrx^ΔE2^ + vehicle, 64.8 ± 7.7 s; Atrx^ΔE2^ + SA4503, 24.8 ± 2.8 s).

### 2.4. Treatment with SA4503 Increases the BDNF (Brain-Derived Neurotrophic Factor) Protein Level in mPFC of Atrx^ΔE2^ Mice

**Figure 4:**
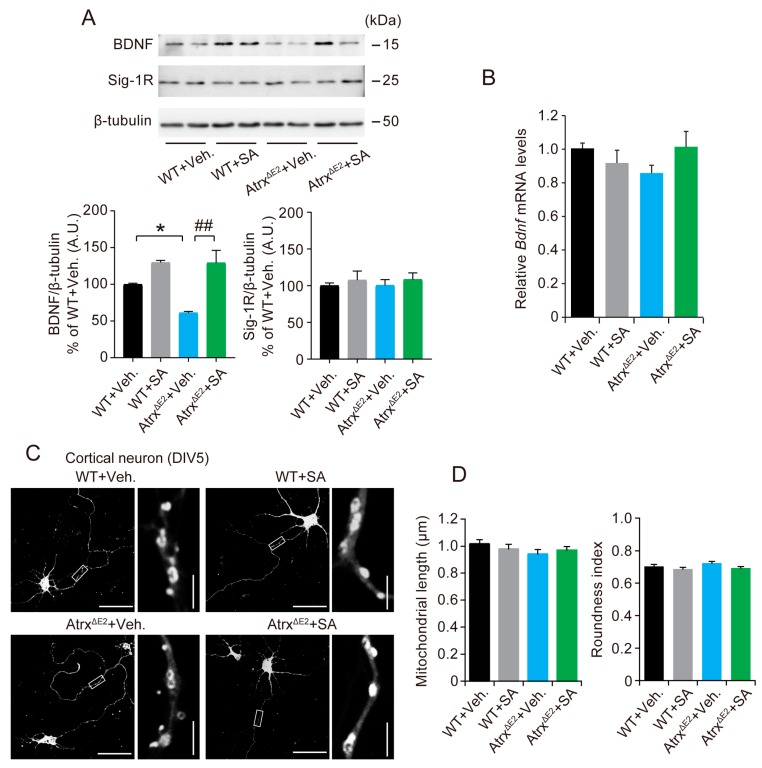
Treatment with SA4503 increases BDNF protein level in Atrx^ΔE2^ mouse brain. (**A**) Representative immunoblot of mouse medial prefrontal cortex lysates probed with the indicated antibodies (top panel). Densitometry analysis of indicated proteins to β-tubulin (arbitrary units; (A.U.)) (bottom panel). * *p* < 0.05, versus vehicle-treated WT mice; ## *p* < 0.01, versus vehicle-treated Atrx^ΔE2^ mice by one-way ANOVA with post hoc Tukey’s test; F (3, 16) = 13.7 in BDNF; *n* = 5 mice each. (**B**) Real-time RT-qPCR showing total *BDNF* mRNA (using exon IX primers) expression in mouse medial prefrontal cortex lysates. *BDNF* mRNA were determined relative to *Gapdh* mRNA. *n* = 5 mice for each group. The experiments were repeated two times with similar results. (**C**) Representative images of cultured cortical neurons at DIV5 with the mitochondrial-specific marker, MitoTracker Red. Images in the right panels are enlarged from the corresponding boxed areas. Scale bars: left panels, 50 µm; right panels, 3 µm. (**D**) Data show the mitochondrial length (left) and the roundness index (right); *n* = 20 cells each. Abbreviations: Veh., vehicle; SA, SA4503.

Next, we explored whether the expression of the BDNF protein was altered in Atrx^ΔE2^ mice. We observed significant decreases in the levels of the BDNF protein in the mPFC of Atrx^ΔE2^ relative to WT mice without changes in the mRNA level; treatment with SA4503 reversed the decrease (Figure 4A,B). However, there was no significant change in NR1, a down stream target of BDNF, or in the Sig-1R protein expression between groups. Sig-1Rs act on restoring Ca^2+^ transferring into mitochondria and ATP productions in Chinese hamster ovary (CHO) cells [33] and neuroblastoma Neuro-2a cells [34], as well as changes in mitochondrial morphology in SA4503-treated cardiomyocytes [35]. We investigated mitochondrial morphology in axons of cultured cortical neurons at DIV5 with the mitochondrial-specific marker, MitoTracker Red. There were no significant changes in mitochondrial length or the roundness index between the groups (Figure 4D), suggesting that Sig-1Rs have no effect on either mitochondrial degradation or enhanced mitochondrial fusion in *Atrx* depletion or cultured cortical neurons.

## 3. Discussion

In this study, we demonstrated that postnatal activation of Sig-1Rs in Atrx^ΔE2^ mice ameliorates some of the intellectual disability-related abnormalities present at the levels of synaptic morphology and behavior. We have also provided evidence that activation of Sig-1Rs enhanced BDNF protein expression in mPFC of Atrx^ΔE2^ mice. These results indicate that the pharmacological rescue of abnormal synaptic morphology and behavior in the adult ATR-X mouse model and identified postnatal Sig-1R activation as a potential therapeutic strategy for countering the various debilitating symptoms of intellectual disability, including those of ATR-X syndrome.

Dendritic spines can assume various shapes by which they are classified; these include filopodia, thin, stubby, or mushroom-like [36,37]. Small spines (filopodia and thin) change their form rapidly, either disappearing or growing into large spines during intense neuronal activity in the mouse brain [38,39,40,41]. Small spines are often short-lived, usually representing weak or silent synapses [42]. Such observations suggest that structural alterations of small spines underlie adaptive and learning processes [43]. Conversely, large spines (stubby and mushroom) are relatively stable and survive for long periods of time: more than a month [40] or even for a year [41] in the mouse cortex in vivo. This observation suggests that memory is maintained in a structural form for extended periods in the brain. The link between spine abnormalities, increases of small spines and intellectual disability has been reported in Down, Fragile X, and Rett syndromes [17,18]. The present report found diminished axonal development and increases of dendritic filopodia in cultured Atrx^ΔE2^ neurons at early stage DIV5 (Figure 1). Moreover, we have demonstrated that neurons in Atrx^ΔE2^ mice exhibit longer, thinner dendritic spines relative to WT neurons at DIV24. This finding is consistent with an in vivo study of adult Atrx^ΔE2^ mice [15], suggesting that the immature patterning of dendritic spines occurs during postnatal brain development of Atrx^ΔE2^ mice. Notably, short-term treatment with SA4503 (48 h) reversed the axonal degeneration and dendritic spine immaturity in cultured Atrx^ΔE2^ neurons in both DIV5 and DIV24 (Figure 1 and Figure 2). The short-term in vivo treatment with SA4503 (i.p., daily from Postnatal Day 70 to Postnatal Day 84) to Atrx^ΔE2^ mice also rescued decreased cognitive deficits (Figure 3). These results indicate that the maturation of dendritic spines provides a potential avenue for post-diagnostic therapy in ATR-X syndrome, as even in mature neurons the size and shape of spines can change. The plastic nature of dendritic spines might be a key component of cognitive function therapy that address intellectual disabilities. In addition, we reported that aberrant expression of Xlr3b is involved in cognitive dysfunction in Atrx^ΔE2^ mice [16]. Interestingly, overexpression of Xlr3b shows abnormalities in dendrite branching, spine development, and synapse formation in Layer II–III neurons of the mouse cerebral cortex [44]. In the future, we will examine that involvement of Xlr3b in abnormality of spine formation in ATR-X syndrome.

Sig-1R plays an important role in the development and stability of dendritic spines. Knockdown of Sig-1Rs by small interfering RNA (siRNA) causes a deficit in the formation of dendritic spines in rat hippocampal primary neurons [45]. In addition, SA4503 induces the upregulation of BDNF protein in the rat hippocampus [46]. SA4503 does not merely increase the total amount of BDNF but potentiates the posttranslational processing of BDNF proteins related to protein secretion in rat neuroblastoma B104 cells [47]. In addition, knockdown of Sig-1Rs decreases the release of mature BDNF without significantly affecting protein synthesis of BDNF [47]. BDNF promotes dendritic and axonal growth, increases dendritic spine density, and enhances synaptic plasticity [27,28,29]. The human *BDNF* gene product having a methionine substitution for valine at codon 66 (Val66Met) impairs BDNF trafficking and its activity-dependent release, thereby impairing cognition in the general population [48]. Dendritic complexity is reduced in dentate granule cells of Val66Met knock-in mice [49], indicating that proper secretory trafficking of BDNF is essential to dendritic spine development and plasticity. We found significant decreases of BDNF protein levels in the mPFC of Atrx^ΔE2^ mice, and treatment with SA4503 recovered the BDNF protein levels without changing the levels of mRNA level (Figure 4). Although we could not reveal the exact molecular mechanism underlying decreases of BDNF protein level in Atrx^ΔE2^ brain, our results indicate that the activation of Sig-1Rs leads to an increased release of BDNF, resulting in the recovery of synaptic morphology and behavior abnormalities of Atrx^ΔE2^ mouse. Similarly, we could not find an association between Ca^2+^/calmodulin-dependent protein kinase II (CaMKII) signaling pathway and Sif-1R/BDNF changes, suggesting that multiple signaling pathways may contribute to altered spine morphology in Atrx^ΔE2^ mice. 

Sig-1R is a stress- and ligand-regulated, endoplasmic reticulum (ER) chaperone protein [50]. Its ability to modulate the actions of neurotransmitter receptors and ion channels explains its involvement in neuroprotection, neuroplasticity, and the release of neurotransmitters [26], especially the activation of the Sig-1R enhanced BDNF secretion in primary rat astrocytes [51], in the mouse striatum [52] and in rat neuroblastoma B104 cells [47]. In addition, Sig-1R dissociates from the mitochondrion-associated ER membrane by Sig-1R agonist and redistributes widely within the cell [26]. The agonist-induced redistribution of Sig-1R is linked to an increased intracellular trafficking of BDNF required for brain repair in rat models of stroke [53] and in 6-hydroxydopamine lesion mouse model of Parkinson’s disease [52]. Interestingly, a Sig-1R-active antidepressant, imipramine, potentiates BDNF-induced activation of the phospholipase C-gamma (PLC-gamma)/inositol 1,4,5-trisphosphate (IP_3_)/Ca^2+^ pathway that triggers glutamate release from cortical neurons [54]. The other selective Sig-1R agonist, PRE084 (2-(4-morpholinethyl)1 phenylcyclohexanecarboxylate), elevates the expression of glial cell-derived neurotrophic factor (GDNF) protein in astrocytes after root avulsion injury in rats [55]. The prototypic Sig-1R agonist, (+)-pentazocine, as well as the Sig-1R-active antidepressants, imipramine and fluvoxamine, potentiate nerve growth factor (NGF)-induced neurite sprouting of PC12 cells [56]. Sig-1Rs also potentiate epidermal growth factor (EGF) signaling toward neuritogenesis in PC12 cells [57]. Thus, the synergistic effects of some growth factors through Sig-1Rs may relate to the observed amelioration of dendritic spine abnormalities and cognitive dysfunction in Atrx^ΔE2^ mice. 

In conclusion, although future studies will be necessary to further characterize the precise molecular mechanisms of the role of BDNF in ATR-X syndrome, our findings clearly demonstrate that Sig-1R activation can exert actions that reverse several key neuronal and behavioral symptoms of ATR-X syndrome. Taken together, these results indicate a primary, and potentially causal, role for defects in spine morphogenesis in intellectual disability. By examining common clinical phenotypes correlated to spine and synaptic abnormalities in intellectual disability, we can elucidate causalities of dysgenesis and identify potential targets for therapeutic intervention.

## 4. Materials and Methods

### 4.1. Animals

Mice (C57BL/6J) were housed under climate-controlled conditions with a 12-h light/dark cycle and were provided with standard food and water ad libitum. Animal studies were conducted in accordance with the Tohoku University institutional guidelines. Ethical approval (9 November 2015) was obtained from the Institutional Animal Care and Use Committee of the Tohoku University Environmental and Safety Committee. The generation of homozygous Atrx^ΔE2^ mice is described in a previous study [14]. In brief, to construct an *Atrx* targeting vector, a 2-kb EcoRV-SpeI genomic fragment of the *Atrx* gene and a 5.5-kb XhoI-BamHI fragment were used as short and long homologous regions, respectively. The IRES-beta-geo cassette with a splicing acceptor site was used to replace the SpeI-XhoI fragment, which contains exon 2. The targeting vector was transfected into J1 ES cells, and G418-resistant clones were selected. Homologous recombinants were identified by Southern blot analysis. Atrx^ΔE2^ mice were backcrossed six generations onto C57BL/6J. For RT-qPCR, immunoblotting and behavioral analyses, male mice at 10–12 weeks of age were used. 

### 4.2. Cell Culture and Transfection

Primary cultures of neurons were obtained using previously described methods [16]. Briefly, cortical tissue was dissected from Embryonic-Day-18 mice and dissociated by trypsin treatment and trituration through a Pasteur pipette. Neurons were plated on coverslips coated with poly-l-lysine in MEM (Invitrogen, Carlsbad, CA, USA). After cell attachment, coverslips were transferred to dishes containing a glial cell monolayer and maintained in Neurobasal Medium (Invitrogen) containing 2% B27 supplement (Invitrogen) and 1% GlutaMax (Invitrogen). Cytosine β-d-arabinofuranoside (5 µM; Sigma-Aldrich, St. Louis, MO, USA) was added to cultures at 3 days in vitro (DIV3) after plating to inhibit glial proliferation. Primary neurons were transfected with GFP (green fluorescent protein) expression vector using electroporation at DIV0. For electroporation, mixture of the single cell suspension with plasmid DNA made its final concentration reach 3 × 10^5^ cells and 5 µg plasmid DNA (1 µg/µL) in 100 µL Opti-MEM® medium. Two pulses of 275 V, 0.5 ms each at 50 ms intervals, were delivered through electroporation cuvettes (2 mm gap) with an electroporator (NEPA21; NEPAGENE, Chiba, Japan). Cortical neurons were harvested at DIV5 and DIV24. These time points correspond to neuronal developmental stages of axon formation and dendrite outgrowth (stage 4) and synaptogenesis and maturation (stage 5), respectively [58,59]. Differential distributions of axons and dendrites could be detectable from DIV3, because axons elongate 5–10 times faster than dendrites; longer neurites could thus be identified as axons [60].

### 4.3. Drugs

**Figure 5:**
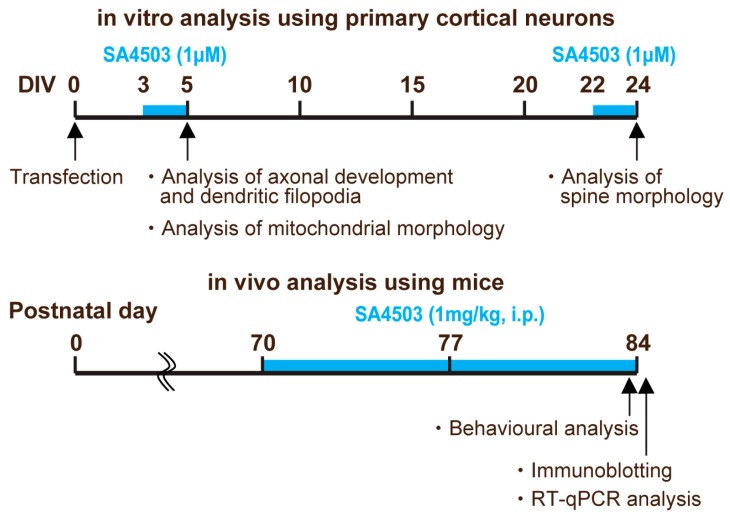
Diagrams of the experimental schedule. In in vitro analysis using primary cortical neurons, SA4503 (1 µM, dissolved in distilled water) was treated for 48 h in cultured cortical neurons in both DIV5 and DIV24. In in vivo analysis using mice, we administered SA4503 over the course of two weeks (1 mg/kg, intraperitoneally (i.p.) daily from postnatal day (P) 70 to P84) to Atrx^ΔE2^ mice and subsequently assessed memory-related behaviors and biological studies.

SA4503 was synthesized in the Laboratory of Medicinal Chemistry, Zhejiang University according to previously described methods [61]. SA4503 (1 µM, dissolved in distilled water) was treated for 48 h in cultured cortical neurons in both DIV5 and DIV24. Mice were randomized into four groups corresponding to differential drug administration as follows: We administered SA4503 over the course of 2 weeks (1 mg/kg, intraperitoneally (i.p.) daily from postnatal day (P) 70 to P84) to Atrx^ΔE2^ mice and subsequently assessed memory-related behaviors. In studies on learning and memory, effective doses of SA4503 are commonly 1.0 mg/kg or less for rodents [62,63]. A diagram of the experimental schedule is given as Figure 5.

### 4.4. Antibodies

For Western blot analysis, we used the following: anti-BDNF antibody (1:1000, AB1779SP, Millipore, Burlington, MA, USA); anti-Sig-1R antibody (1:1000, ab53852, Abcam, Cambridge, UK); anti-β-Tubulin antibody (1:1000, T0198, Sigma-Aldrich); goat anti-mouse IgG (H + L) and human ads-HRP (1:500, 1031-05, SouthernBiotech, Birmingham, AL, USA); goat anti-rabbit IgG (H + L); and mouse/human ads-HRP (1:500, 4050-05, SouthernBiotech). For immunohistochemistry, we used the following: living colors® Full-Length GFP polyclonal antibody (anti-GFP) (1:300, 632592, Clontech, Mountain View, CA, USA); donkey anti-Rabbit IgG (H + L) highly cross-adsorbed secondary antibody; and Alexa Fluor 488 (1:500, A21206, Invitrogen).

### 4.5. RT-qPCR Analysis

Total RNA was purified from mice brains using an RNeasy Mini Kit (Qiagen, Hilden, Germany) according to the manufacturer’s protocol. RNA was reverse transcribed into single-stranded cDNA using an oligo(dT) primer (Promega, Madison, WI, USA) and Moloney murine leukemia virus reverse transcriptase (Invitrogen) and then subjected to RT-PCR with gene-specific primers. RT-qPCR analysis was performed as described previously [16] in 48-well plates (Mini Opticon Real-Time PCR system, Bio-Rad, Hercules, CA, USA) using iQ SYBR Green Supermix 2× (Bio-Rad). Gene expression was assessed using the differences in normalized CT (cycle threshold) (∆∆*C*_t_) method after normalization to GAPDH. Fold change was calculated by 2^−∆∆*C*t^. The following primers were used for RT-qPCR:

Mouse Bdnf in exon IX (FW) (5′-AAGGACGCGGACTTGTACAC-3′)
Mouse Bdnf in exon IX (RV) (5′-CGCTAATACTGTCACACACGC-3′)
Mouse Gapdh (FW) (5′-TGTGTCCGTCGTGGATCTGA-3′)
Mouse Gapdh (RV) (5′-CACCACCTTCTTGATGTCATCATAC-3′).

### 4.6. Immunoblotting

Immunoblotting analysis was performed as previously described [16]. Briefly, tissues were homogenized in a buffer containing 50 mM Tris-HCl (pH 7.5), 0.5% Triton X-100, 0.15 M NaCl, 4 mM EDTA, 4 mM EGTA, 1 mM Na_3_VO_4_, 50 mM NaF, 1 mM dithiothreitol and protease inhibitors (2 µg/mL pepstatin A, 1 µg/mL leupeptin, and 2 µg/mL trypsin inhibitor), and then treated with Laemmli’s sample solution and boiled for 3 min. Bradford protein assay was used to measure the concentration of total protein in a sample. Equivalent amounts of protein (5 µg) were electrophoresed on SDS-PAGE and proteins were then transferred to an Immobilon polyvinylidene difluoride membrane. The procedure of immunoblotting analyses were carried out as previously described [16]. For housekeeping protein, β-tubulin was used as loading control.

### 4.7. Immunohistochemistry

Immunohistochemistry was performed as described previously [16]. Briefly, fixed cells with 4% paraformaldehyde in Phosphate buffered saline (PBS) were treated with PBS containing 0.1% Triton X-100 for 30 min. Samples were incubated overnight at 4 °C with enhanced green fluorescence protein (EGFP) antibody and then washed in PBS and incubated with the secondary antibody, Alexa 488-conjugated donkey anti-rabbit (1:500; A-21206, Invitrogen). Fluorescence images were analyzed by confocal laser scanning microscopy (LSM700, Carl Zeiss). 

### 4.8. Spine Morphological Analysis

Spine-head diameters were measured along an axis perpendicular to the spine neck, approximately in the middle of the spine head. Spine length was calculated as the radial distance from the tip of the spine head to the dendritic shaft. The spine diameter corresponded to the maximum dimension of the spine head. In cases where the spine head showed irregular morphology, or if the spine-head maximum diameter was not perpendicular to the neck, we measured the maximum head diameter. Spines were categorized according to a previously described method [64] along an immature- to mature-appearing spine continuum. Measurements were obtained using ImageJ (National Institutes of Health freeware). 

### 4.9. Analysis of Mitochondrial Morphology

Analysis of mitochondrial morphology was performed as described previously [65]. Briefly, cells were treated with MitoTracker™ Red CMXRos (final concentration 350 nM, M7512, Invitrogen) for 15 min and then treated with 4% paraformaldehyde in PBS (phosphate buffer saline). After images were acquired by confocal laser scanning microscopy (LSM700, Carl Zeiss, Oberkochen, Germany), they were analyzed using ImageJ software. ImageJ counts each signal and simultaneously determines the pixel area, perimeter, and maximum length for each signal. From these measurements, ImageJ calculates a score for roundness index: roundness index = 4π × (area/perimeter^2^).

### 4.10. Behavioral Analysis

Mice were subjected to behavioral tests including the Y-maze, novel object recognition and Barnes maze tasks. The videotapes for all behavioral analyses were scored by a trained observer blind to the genotype and treatment. In the Y-maze task using apparatus consisted of three identical arms (50 × 16 × 32 cm^3^) of black Plexiglas, spontaneous alternation behavior in a Y-maze was assessed as a task of spatial reference memory. An alternation was defined as consecutive entries into all three arms. The maximum number of alternations was defined as the total number of arms entered minus two, and the percentage of alternations was calculated as actual alternations/maximum alternations × 100. The total number of arms entered during the session was also determined. The novel object recognition task used an open-field box (35 × 25 × 35 cm^3^). During the acquisition phase, two objects of the same material were placed symmetrically in the center of the chamber for 10 min. After 24 h, one object was replaced by a novel object, and exploratory behavior was analyzed again for 5 min. Exploration of an object was defined as rearing on the object or sniffing it at a distance of <1 cm, touching it with the nose or both. Discrimination of spatial novelty was assessed by comparing the difference between exploratory contacts of novel and familiar objects and the total number of contacts with both, making it possible to adjust for differences in total exploration contacts. The Barnes maze consists of a circular platform (92 cm in diameter) with 20 holes (hole diameter: 5 cm) along its perimeter. At the beginning of each trial, the mouse was placed in the middle of the maze in a cylindrical start chamber (7.5 cm). After 10 s, the chamber was lifted and the mouse was free to explore the maze. The test ended when the mouse entered the goal tunnel or after 3 min elapsed. Immediately after the mouse entered the tunnel, the mouse was allowed to stay in the tunnel for 1 min. Mice were trained for two trials per day for 4 days. Errors and search time required to escape into the tunnel were recorded as the test parameters; errors were defined as nose pokes and head deflections over any hole that did not lead to a tunnel.

### 4.11. Statistical Analysis

All values are expressed as means ± SEM. Statistical significance for differences among groups was tested by one-way analysis of variance (ANOVA) with post-hoc Tukey’s multiple comparison test or two-way ANOVA with post-hoc Bonferroni’s multiple comparison test. Cumulative percentage of spines was tested by the Kolmogorov–Smirnov test. *p* < 0.05 was considered significant. All statistical analyses were performed using GraphPad Prism 7 (GraphPad Software, La Jolla, CA, USA).
